# Blood and Urinary Biomarkers of Antipsychotic-Induced Metabolic Syndrome

**DOI:** 10.3390/metabo12080726

**Published:** 2022-08-05

**Authors:** Aiperi K. Khasanova, Vera S. Dobrodeeva, Natalia A. Shnayder, Marina M. Petrova, Elena A. Pronina, Elena N. Bochanova, Natalia V. Lareva, Natalia P. Garganeeva, Daria A. Smirnova, Regina F. Nasyrova

**Affiliations:** 1 Institute of Personalized Psychiatry and Neurology, Shared Core Facilities, V.M. Bekhterev National Medical Research Centre for Psychiatry and Neurology, 192019 Saint Petersburg, Russia; 2 Shared Core Facilities Molecular and Cell Technologies, V.F. Voino-Yasenetsky Krasnoyarsk State Medical University, 660022 Krasnoyarsk, Russia; 3 Department of Therapy of Faculty of Postgraduate Education, Chita State Medical Academy, 672000 Chita, Russia; 4 Department of General Medical Practice and Outpatient Therapy, Siberian State Medical University, 634050 Tomsk, Russia; 5 International Centre for Education and Research in Neuropsychiatry, Samara State Medical University, 443099 Samara, Russia

**Keywords:** metabolic syndrome, antipsychotic-induced metabolic syndrome, personalized psychiatry, antipsychotics, serum biomarkers, urinary biomarkers, personalized metabolomics

## Abstract

Metabolic syndrome (MetS) is a clustering of at least three of the following five medical conditions: abdominal obesity, high blood pressure, high blood sugar, high serum triglycerides, and low serum high-density lipoprotein (HDL). Antipsychotic (AP)-induced MetS (AIMetS) is the most common adverse drug reaction (ADR) of psychiatric pharmacotherapy. Herein, we review the results of studies of blood (serum and plasma) and urinary biomarkers as predictors of AIMetS in patients with schizophrenia (Sch). We reviewed 1440 studies examining 38 blood and 19 urinary metabolic biomarkers, including urinary indicators involved in the development of AIMetS. Among the results, only positive associations were revealed. However, at present, it should be recognized that there is no consensus on the role of any particular urinary biomarker of AIMetS. Evaluation of urinary biomarkers of the development of MetS and AIMetS, as one of the most common concomitant pathological conditions in the treatment of patients with psychiatric disorders, may provide a key to the development of strategies for personalized prevention and treatment of the condition, which is considered a complication of AP therapy for Sch in clinical practice.

## 1. Introduction

Metabolic syndrome (MetS) combines a set of pathological clinical and metabolic data, describing key links in the pathogenesis of cardiovascular diseases (CVD). MetS includes visceral obesity, insulin resistance (IR), high blood pressure (BP), elevated triglycerides (TG), and low serum high-density lipoprotein cholesterol (HDL-C). The main MetS criteria are presented by the National Cholesterol Education Program in the Adult Treatment Panel (ATPIII) [1] and the Adapted Adult Treatment Panel (ATPIII-A) proposed by the American Heart Association [2], and by the International Diabetes Federation (IDF), which emphasizes the importance of measuring waist circumference as a MetS characteristic [3]. The MetS criteria are defined by the following indicators, according to all three of the above-mentioned panels [1,2,3]: WC size: men > 102 cm, women > 88 cm (exception: IDF criteria: men > 94 cm, women > 80 cm); BP: ≥130/≥85 mm Hg; serum HDL < 40 mg/dL (1.04 mmol/L); serum TG ≥ 150 mg/dL (1.7 mmol/L); fasting serum glucose ≥ 100 mg/dL (5.6 mmol/L; exception: ATPIII criteria: ≥110 mg/dL (6.1 mmol/L).

In the general population, MetS is associated with an increased (four-fold) relative risk of developing diabetes mellitus (DM) [4] and an increased (two-fold) risk of developing coronary heart disease (CHD), stroke, and premature death [5]. As a consequence, MetS has been proposed as an alternative to the Framingham estimate of the likelihood of CVD occurring over the next 10 years [6]. The same results of the influence of MetS as a predictor of the development of CVD were also established in patients with schizophrenia (Sch) [7].

Sch is a common, socially significant mental disorder associated with premature mortality and reduced life expectancy [8,9]. Epidemiological studies show that the life expectancy of patients with serious mental disorders, including Sch, is reduced by 7–24 years [10]. Previously, the increased risk of mortality in Sch was explained by the high frequency of suicide [11]. In recent years, studies have demonstrated the leading role of concomitant somatic diseases in patients with Sch, while risk factors for the development of CVD have become the main cause of death in these patients [8,12]. Therefore, timely diagnosis and assessment of risk factors for the development of MetS and CVD should be one of the priority strategies in the management of patients with Sch [12].

According to the ATPIII criteria, MetS prevalence is 18.4% among men and 14.4% among women in Europe, and 28.8% among men and 31.8% among women in South Asian countries, while this rate among the general population is 15.7% in Taiwan and 23.7% in the USA, respectively [13]. In a systematic review and meta-analysis by Mitchell et al. [14], the results were analyzed of 126 studies in 77 publications involving 25,692 patients with Sch from 2003 to 2011. The authors showed that 64.0% of patients were men with a mean duration of illness of 10.4 years. The overall prevalence of MetS was 32.5%. Thus, every third patient with Sch suffers from MetS. In accordance with population studies, there were no significant differences in the prevalence of MetS among men and women. When studying individual disorders of MetS, it turned out that one in two patients with Sch was overweight, one in five had significant hyperglycemia, and at least two out of five had lipid disorders [14].

The prevalence of MetS is higher in patients who take antipsychotics (APs) compared to those who do not. Regarding the effect of APs on the development of MetS: the highest prevalence rates were recorded in patients on clozapine therapy (51.9%), and the lowest in those who did not take APs (20.2%) [14].

MetS frequency increases with age (*p*-value < 0.0001). The lowest MetS values were observed in patients who had recently experienced their first psychotic episode. One of the main determinants of a higher MetS risk is a longer disease duration (>7.8 years, *p*-value < 0.0001). The cumulative effect of long-term exposure to APs is the risk of developing CVD and MetS in patients with Sch [14]. In such patients, the greatest prognostic value for the development of MetS is waist circumference, with a sensitivity of 79.4% and a specificity of 78.8% [14].

All this suggests that the risk of developing MetS in patients with Sch is a key factor affecting long-term health in these patients [14]. Due in part to concerns about the development of AP-induced adverse drug reactions (ADRs) of the metabolic spectrum with new APs, there is exponential growth in the number of studies demonstrating a high prevalence of DM 2 and prediabetic conditions such as MetS in patients with Sch [15]. MetS can be induced by APs, both of the first and second generations, and the criteria for AP-induced MetS (AIMetS) do not differ from those for MetS [16].

Predicting the development of MetS in patients with Sch continues to attract a high level of interest in the psychiatric community, as it makes it possible to identify high-risk groups and prevent the progression of some of the main causes of comorbid somatic pathologies and mortality in this category of patients. Despite the development of new APs, the problem of AIMetS and MetS in patients with mental disorders, in particular, with Sch, has not been solved. At the same time, early diagnosis of MetS is especially important. According to the MetS criteria of various associations, urinary biomarkers, unlike serum biomarkers, are not included in the list of MetS diagnostic criteria; however, urine is very concentrated and may also contain MetS markers, making early diagnoses possible. Laboratory diagnostics of AIMetS is mainly based on the study of biomarkers in plasma and serum, much less frequently in urine. The most important plasma and serum markers are better understood, although the urinary AIMetS biomarkers have been actively studied over the past few years. Such study is promising, since biomaterial sampling is not invasive, which seems convenient for patients with mental disorders.

**Figure 1:**
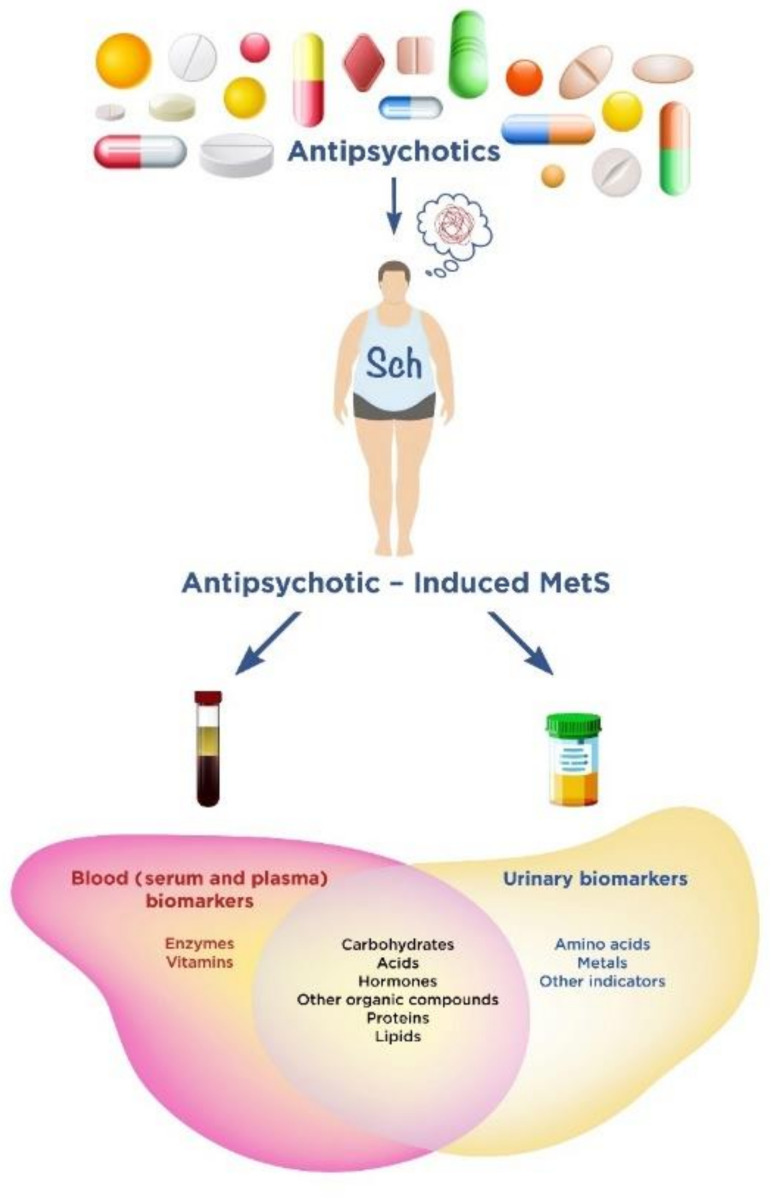
Potential Blood (Serum and Plasma) and Urinary Biomarkers of Antipsychotics—Induced Metabolic Syndrome (AIMetS) in Patients with Schizophrenia (Sch).

The main purpose of our narrative review is to summarize the accumulated data on serum, plasma and urinary AIMetS biomarkers in patients with Sch, which have prospects for application in clinical practice. We divided the analyzed potential AIMetS biomarkers into groups according to their chemical structure (Figure 1). We tried to indicate their biological role and participation in various pathogenetic links of MetS, as well as MetS as a comorbid condition in Sch and AIMetS. We understand that these groups are rather arbitrary, because it is difficult to clearly define the biological role of an individual biomarker due to the complexity of the pathogenesis of AIMetS.

## 2. Blood (Serum and Plasma) Biomarkers of Antipsychotic-Induced Metabolic Syndrome

A biomarker is a characteristic that is objectively measured and evaluated to detect disease in patients. Biomarkers act as prognostic tools for classifying and assessing disease progression. They are used to monitor clinical response and positive effects of old (classical) and new therapeutic strategies [17].

Traditionally, laboratory diagnoses of MetS and assessments of CVD risk include analyses of blood (serum or plasma) biomarkers, i.e., total cholesterol (TC), TG, HDL-C, low-density lipoprotein cholesterol (LDL-C), insulin and C-peptide [18].

The mechanisms of MetS development continue to be studied, with many studies indicating that it is closely associated with inflammation [19], insulin resistance [20], vascular endothelial dysfunction [21], renal dysfunction [22], oxidative stress [23] and liver dysfunction [24]. It is promising and reasonable to use panels of biomarkers for diagnosing MetS. We analyzed studies not only of traditional biomarkers, but also of some new biomarkers associated with inflammatory response and subsequent cardiovascular risk (CVR). MetS has long been associated with dyslipidemia [25].

Metabolic overload causes oxidative stress, a condition where the balance between the production and inactivation of reactive oxygen species (ROS) is disturbed. These substances play an important role in many physiological systems, but under conditions of increased oxidative stress, they contribute to cellular dysfunction [26]. Oxidative stress may play an important role in MetS-related manifestations (atherosclerosis, arterial hypertension (AH), DM 2) [27]. Oxidative stress may be an early event in the pathology of these chronic diseases, and not just a consequence or concomitant process [28]. MetS is accompanied by a chronic, indolent inflammatory state, or meta-inflammation, that is, metabolically induced inflammation [29], or even para-inflammation [30]. Some evidence [31] suggests that inflammation explains some of the risks of CVD, but this does not rule out other mechanisms. It is also worth emphasizing that oxidative stress and mild inflammation are quite closely related pathogenetically [32].

In this study, we considered eight groups of 38 biomarkers: carbohydrates, acids, hormones, other organic compounds, proteins, lipids, enzymes and vitamins.

### 2.1. Carbohydrates

#### Glucose

Elevated fasting glucose (>100 mg/dL) or pharmacotherapy for elevated glucose is one of the MetS criteria [1,2,3]. Most patients with MetS have elevated plasma glucose levels. Fasting plasma glucose levels in the range of 100–125 mg/dL or 2-h postprandial levels of 140–199 mg/dL characterize prediabetes, while DM is defined as fasting glucose >126 mg/dL or postprandial levels > 200 mg/dL [33].

Insulin resistance is the main cause of hyperglycemia in patients with MetS; however, compensatory hyperinsulinemia can lead to normal glucose levels in insulin resistance. When pancreatic beta cell function decreases, compensatory mechanisms fail. Hyperglycemia develops as a later complication and is not the first sign of MetS. Chronic hyperglycemia often leads to microvascular diseases such as chronic kidney disease (CKD) and DM. Diabetic neuropathy may be partially associated with microvascular disease [34]. Microvascular diseases can further accelerate the development of congestive heart failure (HF) and contribute to atherogenesis [35].

There is evidence of a possible common mechanism for the development of diabetes and Sch. More than 38 reports link poor fetal growth with impaired glucose metabolism later in life [36]. Most studies have demonstrated an inverse relationship between birth weight and plasma glucose and insulin levels, type 2 diabetes mellitus and insulin resistance. Low birth weight (due to intrauterine development delay or prematurity) is associated with subsequent neurological and psychiatric problems, including Sch, in children and adolescents [37]. However, the mechanisms underlying these associations have not been sufficiently studied. Nevertheless, acute starvation of the mother in the first trimester of pregnancy during the Dutch famine winter of 1944 led to a two-fold increase in the risk of developing Sch in offspring [38].

Also, the development of MetS may be associated not only with the development of Sch, but also with the use of APs in the treatment thereof. In a blind, randomized, controlled trial involving 157 patients with Sch treated with clozapine, olanzapine, risperidone or haloperidol for 14 weeks, the following results were reported: clozapine, olanzapine, and haloperidol were found to be associated with an increase in glucose levels, while both clozapine and olanzapine are also associated with an increase of serum level of cholesterol [39]. There is also emerging evidence that glucose disturbances may occur shortly after the beginning of the administration of Aps, and that these disturbances may be reversible upon discontinuation of APs, indicating a direct effect of APs on the function of the pancreas [40].

### 2.2. Acids

#### 2.2.1. Sialic Acid

Many acute-phase inflammatory proteins (e.g., haptoglobin, alpha-glycoprotein, fibrinogen, transferrin and complement) are glycoproteins with sialic acid (Sia) as the terminal sugar of the oligosaccharide chain, so serum Sia concentration can be considered a biomarker of acute-phase inflammation response [41]. Serum Sia is a possible risk factor for CVD. The overall level of Sia is also increased in DM2 [42]. Elevated serum and urinary Sia concentrations are strongly associated with the presence of microvascular complications in patients with DM2 [43]. Thus, elevated levels strongly correlate with the presence of MetS [44].

In the general population, a positive correlation has been found between Sia levels and C-reactive protein (CRP) levels [45]. In one study, the authors concluded that this biomarker identifies overweight persons with an inflammatory phenotype who are at high risk of developing MetS [46]. Large-scale epidemiological studies have found a positive correlation between plasma levels of Sia and the risk of CHD. There is evidence that aberrant sialylation of LDL, LDL receptors and blood cells is involved in the pathological process of atherosclerosis.

Sia regulates the immune response by binding to the immunoglobulin-like lectin-binding Sia (Siglecs). The Sia-Siglecs axis is involved in immune inflammation in atherosclerosis [47]. Polysialic acid (polySia) is a unique Sia polymer that spatiotemporally modifies the neural cell adhesion molecule (NCAM) in the embryonic brain. PolySia is an important molecule associated with Sch, regulating intercellular communication through an anti-adhesive effect. PolySia regulates multimolecules such as brain-derived neurotrophic factor (BDNF) and fibroblast growth factor-2 (FGF2) and dopamine. Recently, several studies have reported that PolySia is pathogenetically associated with Sch and other psychiatric disorders [48].

There have been isolated studies that have measured the concentration of Sia acid in patients with Sch; however, due to the small sample size, the findings do not have the required level of reliability [49], and further research is needed on the association of increased serum Sia in patients with Sch, as well as in patients with Sch and MetS, i.e., AIMetS.

#### 2.2.2. Uric Acid

Uric acid is a product of the metabolic breakdown of purine nucleotides. The enzyme xanthine oxidase produces uric acid from xanthine and hypoxanthine, which, in turn, are derived from other purines [50]. Uric acid is a powerful antioxidant [51]. An increase in the level of this biomarker is observed in obesity, and a relationship with MetS is also evident [52]. A longitudinal study showed that elevated serum levels of uric acid is a strong biomarker of MetS [53], and that elevated uric acid is also correlated with several cardiovascular risk factors [54]. Uric acid also tends to worsen insulin resistance and exacerbate hyperlipidemia and fatty liver [55]. It has been suggested that acute uric acid elevation is a protective factor, while chronic uric acid elevation is a risk factor, but there is still no consensus on this issue [56]. Uric acid is not an independent biomarker for predicting MetS, and the question of whether this relationship is causal remains to be explored [57]. It is worth noting that in one study in men with Sch treated with haloperidol, plasma uric acid levels were significantly lower than in the control group. Additionally, after discontinuation of haloperidol, plasma uric acid levels further decreased in patients with Sch. There was also a trend toward a decrease in uric acid levels in patients with relapse compared with clinically stable patients [58]. In a meta-analysis of all case-control studies investigating serum and plasma uric acid levels in subjects with Sch compared to those in healthy subjects, uric acid levels were reduced in subjects having recently experienced their first episode of psychosis, but in the remaining cases, there were no statistically significant differences in uric acid levels between patients with Sch and healthy controls. These data support the clinical evidence that the first psychotic episode is accompanied by an increased response to oxidative stress [59]. Thus, it can be assumed that the complex pathogenetic mechanisms of uric acid involvement in Sch and MetS need to be considered and further investigated for more accurate use as a potential marker of AIMetS.

### 2.3. Hormones

#### 2.3.1. Adiponectin

Adiponectin is a hormone that is synthesized and secreted by white adipose tissue, mainly adipocytes of the visceral region. Its secretion is stimulated by insulin [60]. Adiponectin is involved in the regulation of glucose levels and fatty acid breakdown [61]. Adiponectin concentration is inversely associated with insulin resistance, DM2 and dyslipidemia [62], and an inverse association with MetS has also been confirmed in various studies conducted in different countries [63].

Since adiponectin has anti-inflammatory and anti-atherogenic effects, it is also inversely related to a number of risk factors for cardiac ischemia in obese and overweight people [64,65]. The anti-inflammatory effects of adiponectin were confirmed by the facts that adiponectin is inversely proportional to the level of CRP, interleukin (IL)-6 and IL-10, and that it suppresses the production of tumor necrosis factor-a (TNF-a) by macrophages [66]. A prospective study confirmed the role of adiponectin in the development of cardiometabolic disorders in obese patients [67]. Also, scientists are considering screening MetS using adiponectin [68]. High levels of adiponectin correlate with increased insulin sensitivity and glucose tolerance [69].

Clinical and preclinical data have shown that the inability to stimulate the production of adiponectin is associated with metabolic disorders caused by APs. Sch itself is not associated with lower blood levels of adiponectin [70]. In schizophrenic patients with MetS, blood levels of adiponectin are lower compared to patients without MetS, and blood levels of adiponectin decrease as MetS components increase [71]. In patients with Sch taking APs, the level of adiponectin in the blood is also lower than in healthy people [70]. It was found that blood levels of adiponectin increased after a year of administration of risperidone in previously untreated patients with psychotic disorders [72]. Blood levels of adiponectin were also found to increase in patients with Sch treated with risperidone for 3 and 12 months, respectively [73].

In patients with Sch taking olanzapine for more than 3 months, there is a decrease in the level of adiponectin in the blood [73,74]. In patients with Sch receiving clozapine for a long time, the level of adiponectin in the blood is lower than in healthy people [75]. In patients with Sch treated with clozapine for at least 3 months, blood levels of adiponectin are negatively associated with weight gain and metabolic biomarkers after chronic administration of clozapine [76].

Thus, it is necessary to take into account changes in the concentration of adiponectin in the blood of patients with Sch, as well as for those receiving and not receiving APs treatment, since not all APs are able to induce MetS, and a decrease in adiponectin is not always observed when taking Aps. This issue requires further study and research.

#### 2.3.2. Aldosterone

Aldosterone is a mineralocorticoid hormone that is involved in the regulation of sodium balance. It is elevated in patients with MetS [77]. An increase in aldosterone may exacerbate the development of MetS-related hypertension [78]. Also, aldosterone excess may predispose or exacerbate metabolic and cardiovascular complications in patients with obstructive sleep apnea [79].

There is also evidence of an increase in renin activity and plasma aldosterone levels during treatment with haloperidol as a dopamine antagonist; however, it should be noted that MetS was not studied in these patients [80].

#### 2.3.3. Chemerin

Chemerin is an adipokine that has chemoattractant activity. This adipokine is secreted as an inactive proprotein [81]. Chemerin and its receptors are mainly expressed in adipose tissue. Chemerin, as an adipokine, was found to be involved in the pathophysiology of MetS. Plasma concentrations of chemerin were closely associated with body mass index (BMI), TG and BP [82]. Recent studies have demonstrated high plasma levels of chemerin in patients with incipient MetS, which indicates the involvement of this adipokine in the pathogenesis of MetS and its role as an early metabolic biomarker [83]. Also, chemerin is considered a biomarker of atherosclerosis in MetS, and an elevated level thereof is considered an independent predictive biomarker of cardiac ischemia in patients with MetS [84]. Chemerin and adiponectin may be reciprocally involved in the development of MetS [85]. Clinical data consistently indicate that circulating chemerin levels are elevated in patients with obesity, DM and CVD [86].

In a rat study, in addition to inducing hyperphagia and weight gain associated with increased AMP-activated protein kinase activity and decreased H1 receptor expression in the arcuate nucleus, the administration of olanzapine also increased hypothalamic chemerin, presumably in response to weight gain [87].

#### 2.3.4. Ghrelin

Ghrelin is a peptide of 28 amino acids which is involved in the regulation of appetite, as well as energy balance [88]. It is produced predominantly by enteroendocrine cells of the gastric fundus [89], and has a wide range of effects on various tissues and organs of the human body, including stimulation of the secretion of lactotrophs and corticotrophs, effects on the function of the gastrointestinal tract and pancreas, regulation of insulin secretion, regulation of glucose and lipid metabolism, influence on behavior and sleep, and regulation of the functions of the cardiovascular system [90]. Ghrelin characteristically increases food intake and body weight due to its orexigenic, adipogenic and somatotropic properties [91].

Blood (plasma) levels of ghrelin decrease in obesity and increase in anorexia nervosa in humans [92]. A progressive decline in basal ghrelin levels is associated with an increase in BMI [93]. Low ghrelin concentrations are also associated with higher MetS prevalence [94]. The total levels of ghrelin in plasma in obese patients with MetS are lower compared to those in non-obese patients. Recently, no significant difference in ghrelin concentrations was found between postmenopausal women with and without MetS [95].

An analysis of five prospective and three cross-sectional studies suggested that the concentration of ghrelin in the blood in the treatment of APs is biphasic and depends on the duration of treatment, i.e., it decreases in the early stages and increases in the late periods [96].

#### 2.3.5. Insulin

Insulin is a protein hormone. It is produced in the beta cells of the islets of Langerhans of the pancreas. It has a multifaceted effect on metabolism in almost all tissues. The main action of insulin is the regulation of carbohydrate metabolism, in particular, the utilization of glucose in the body [97]. The level of insulin in the blood increases by 30–70% in Sch patients with chronic administration of clozapine or olanzapine [98]. It has also been reported that olanzapine-treated patients have significantly higher mean insulin levels than those treated with conventional Aps, despite having the same BMI, suggesting a possible effect of olanzapine on pancreatic insulin secretion [99]. Because hyperinsulinemia appears to be concentration-dependent in both clozapine-treated and olanzapine-treated patients, lowering the doses of these drugs may reduce hyperinsulinemia in affected patients [100]. Insulin is a hormone that can cause weight gain by directly affecting adipose tissue and appetite through hypoglycemia. Reducing the dose of clozapine or olanzapine may lead to weight loss in patients with AP-induced obesity by reducing the severity of hyperinsulinemia [101].

#### 2.3.6. Leptin

Leptin (LEP) is a proteohormone involved in the modulation of appetite, regulation of energy balance and weight [102]. A loss of LEP leads to obesity [103]. In particular, LEP acts on the hypothalamus to reduce food intake and increase energy expenditure [104]. During periods of energy balance (maintenance of body weight), blood LEP concentrations reflect the total amount of fat in the human body [105]. LEP is involved in peripheral insulin resistance, impairs the action of insulin on insulin-responsive cells and may induce insulin resistance by affecting insulin secretion. In the human liver, leptin has been shown to attenuate the number of insulin-induced actions that ultimately lead to insulin resistance [106]. Subjects with MetS have higher LEP levels compared to individuals without MetS [107]. Various authors have stated that plasma LEP is a significant predictor of MetS risk [108].

Clozapine and a number of other antipsychotics have been shown to increase serum levels of leptin, which may cause AP-induced weight gain. The SNV rs7799039 (G2548A) in the promoter region of the *LEP* gene was found to be associated with the obesity phenotype. This SNV affects *LEP* expression and increases the level of LEP secretion by adipose tissue adipocytes. This SNV was found to be associated with AP-induced weight gain after 10 weeks of risperidone or chlorpromazine administration in Chinese patients with Sch. In addition, the homozygous AA genotype may be a genetic biomarker of AIMetS [109].

#### 2.3.7. Omentin

Human omentin is a 313 amino acid peptide [110] originally identified in omental fat. It is reduced in obese patients [111], and is downregulated in association with metabolic disorders with obesity [112]. Circulating omentin levels are negatively correlated with metabolic risk factors. Omentin has been suggested as a biomarker for MetS [113] and has also been reported as a biomarker for endothelial dysfunction in patients with MetS [114].

In a study comparing plasma concentrations of omentin in patients with Sch and healthy individuals, it was found that omentin levels were significantly lower in patients with Sch. It was also found that omentin concentration was negatively correlated with the severity of the disease, which suggests that fasting omentin levels are lower in patients with more severe pathologies [115].

#### 2.3.8. Parathyroid Hormone

Elevated plasma levels of parathyroid hormone (PTH) [116] have been associated with MetS and each of its individual components [117]. PTH elevation is considered a compensatory mechanism for low 25(OH)D. In most cases, PTH, but not vitamin D, is associated with MetS [118]. The role of PTH as a link between PTH levels and biomarkers of inflammation in the adult population of the USA has been confirmed [119]. High PTH levels are associated with a higher risk of cardiorenal MetS [120]. However, some authors argue that serum 25(OH)D, but not PTH, was significantly related to the levels of MetS and several of its component [121].

In a prospective study of patients with psychiatric disorders who took APs, a trend of decreasing PTH after a week of taking Aps was found. In that study, the lack of significance of the indicators was due to the small sample [122].

#### 2.3.9. Testosterone

There is evidence that testosterone is involved in stimulating glucose uptake, glycolysis, stimulating glucose utilization and mitochondrial oxidative phosphorylation. Testosterone is also involved in the homeostasis of lipids in tissues such as the liver, adipose tissue and skeletal muscle [123]. Testosterone levels decrease with MetS [124]. Intervention studies have shown beneficial effects of testosterone on MetS components [125] and improved body composition [126].

In a cross-sectional study of 190 patients with Sch taking APs, it was found that the decrease in testosterone levels in patients at high risk of MetS was statistically significant, and the five metabolic indices proposed in the study were negatively correlated with testosterone levels. Thus, testosterone has been proposed as a MetS biomarker in Sch [127].

Also, in a prospective study of testosterone levels in 78 male patients taking APs, at week 3 of the study, with Sch, a negative effect of APs, elevated prolactin levels and a higher BMI on blood testosterone levels was revealed. At week 8, serum testosterone concentrations were also significantly reduced [128].

#### 2.3.10. Thyroid-Stimulating Hormone

An increase in thyroid-stimulating hormone (TSH) levels has been associated with less favorable lipid concentrations [129]. In addition, a slightly elevated serum TSH concentration is associated with increased incidence of obesity [130]. Many publications have noted that higher concentrations of TSH are associated with MetS [131]. However, the risk of CVDs may be high, even with normal TSH [132]. In another publication, higher TSH levels and subclinical hypothyroidism are associated with an increased likelihood of prevalence, but not incidence, of MetS [133].

A strong negative correlation was found between negative symptoms on the Positive and Negative Syndrome Scale (PANSS) and TSH levels [134]. In addition, changes in TG levels are associated with the use of APs [135]. In patients with Sch and MetS, TG levels were significantly higher compared with patients with MetS Sch and patients with MetS in a general hospital [136]. However, a study of 151 patients with Sch receiving long-acting injectable APs demonstrated no change in TSH in patients with or without MetS [137].

### 2.4. Other Organic Compounds

#### Bilirubin

Bilirubin is considered a potentially toxic metabolite of heme catabolism [138]. However, it has well-known antioxidant and anti-inflammatory properties, as evidenced by its ability to scavenge peroxyl radicals, inhibit LDL oxidation and repress the expression of cell adhesion molecules, vascular cell adhesion molecule 1 (VCAM-1) and intercellular adhesion 1 (ICAM-1) in vitro [139]. Moderately elevated bilirubin levels are negatively associated with MetS and other diseases mediated by oxidative stress [140]. Studies in Asian countries have shown that serum total bilirubin in the upper normal range may provide some protection against MetS and reduce future risk of CHD [141]. The fact that highly sensitive CRP (hsCRP) is inversely related to bilirubin also indicates that low bilirubin levels are associated with systemic inflammation [142].

In a study of 131 patients with Sch receiving APs, it was found that the level of direct bilirubin is associated with the diagnosis and course of MetS. These results are similar to those of previous studies in the general population, in overweight individuals and in patients with CVDs [143]. Low levels of direct bilirubin in blood serum are associated with the initial and subsequent diagnosis of MetS, WC and TG criteria at the initial stage, and fasting glucose criteria at follow-up. It was shown that the serum level of indirect bilirubin is statistically significantly associated only with WC criteria at the initial stage. In cases of obesity and MetS, when oxidative stress increases, bilirubin consumption increases, resulting in a decrease in serum bilirubin levels which leads to an increased risk of CVDs, causing endothelial dysfunction, given the antioxidant properties of bilirubin. The relationship between serum direct bilirubin levels and MetS has also been shown in many previous studies [144]. In a sample of 5321 patients, the association between direct bilirubin and the diagnosis of MetS observed during the first and second (after 6 months) visits confirmed this conclusion. Lower levels of direct bilirubin have been associated with more MetS criteria [145]. Risk of MetS is increased two to five times in patients with serum direct bilirubin levels in the lower interquartile percentile (0–75th percentile) compared with those in the uppermost percentile (75th–100th percentile), and an increase in total bilirubin by one standard deviation reduces the risk of MetS by 17%. Similarly, a study by Karadag F. et al. showed that MetS diagnosis was significantly lower in the high bilirubin group [144].

### 2.5. Proteins

#### 2.5.1. Adipocyte Fatty Acid-Binding Protein

Adipocyte fatty acid-binding protein (A-FABP) is a small lipid-binding protein with a molecular weight of 15 kDa. It is an adipokine [146], i.e., it is predominantly produced from adipocytes, but is also produced in macrophages [147] and endothelial cells [148]. The relationship between the diagnosis of MetS and serum A-FABP had a sensitivity of 40% and a specificity of 99% at a protein level of 16 mg/L [149]. Several cross-sectional studies have shown that A-FABP is independently and positively associated with MetS markers, especially those associated with obesity [150]. A-FABP is elevated in patients with familial combined hyperlipidemia [151] and in patients with non-alcoholic fatty liver disease, which is considered a hepatic manifestation of MetS [152]. The level of this protein is also increased in patients with atherosclerosis [153] and CVD [154]. Elevated A-FABP levels are also associated with left ventricular diastolic dysfunction in MetS comorbid obesity, suggesting that A-FABP is associated with cardiometabolic disorders [155]. A high level of A-FABP may be predictive for the development of MetS and CVD [156]. A-FABP decreases after weight loss and statin therapy [157].

#### 2.5.2. C-Peptide

C-peptide is a by-product of insulin synthesis. It is produced in equal molar amounts relative to insulin, primarily by the kidneys, and has a half-life three to four times that of insulin [158]. C-peptide is clinically useful for diagnosing patients with insulin-dependent diseases and may be a biomarker for monitoring MetS in patients with DM 2 [159]. Since there is one molecule of peptide C for every insulin molecule, it is a good marker for assessing the amount of endogenous insulin. C-peptide levels are higher in patients with MetS [160]. The role of C-peptide is considered as a prognostic biomarker of CVD [161].

In a longitudinal study of 112 patients with Sch treated with APs for 8 weeks, C-peptide levels increased significantly in all groups (divided by APs received), but not fasting glucose. Also, the concentration of cholesterol and TG was significantly increased in the clozapine and olanzapine groups. In patients treated with clozapine and olanzapine, fasting insulin and C-peptide levels were higher than in patients treated with risperidone and sulpiride. Among the four APs, the increase in mean BMI from high to low was as follows: clozapine, olanzapine, sulpiride, and risperidone [162]. Similar conclusions were outlined in another study that evaluated, among other things, the level of C-peptide after 7 months of APs treatment in patients with Sch who had not previously received APs. Seven months of APs treatment reduced clinical symptoms but significantly increased BMI, increased the levels of C-peptide (*p*-value = 0.03) and leptin (*p*-value = 0.02) and decreased the level of adiponectin (*p*-value = 0.01) [163].

#### 2.5.3. Ligand CD40

The CD40 ligand (CD40L), a pro-inflammatory mediator, is expressed on CD4+ T cells and is activated by platelets. CD40L is expressed on vascular cells and is increased by MetS [164]. CD40L levels are higher in patients with MetS and cardiac ischemia [165]. The CD40L–CD40 interaction is important in the cascade of inflammatory and proatherothrombotic reactions [166]. This ligand has not yet been studied in patients receiving APs against AIMetS.

#### 2.5.4. Cystatin C

Cystatin-C (cys-C) is a 15 kDa protein that acts as a negative regulator of proatherogenic cysteine proteases [167]. It is also a new biomarker of kidney and cardiovascular function [168]. Since patients with MetS are at high risk of developing renal failure [169], cys-C has been proposed as a sensitive endogenous serum biomarker for changes in glomerular glomerular filtration rate [170]. The level of cys-C is higher in patients with MetS, regardless of creatinine clearance [171]. A progressive increase in cys-C levels, depending on the amount of MetS components [172], indicates an increased risk of CVD [173]. A high level of cys-C may be predictive of MetS, also in the diagnosis of cardiac ischemia [174]. This protein has not yet been studied in patients receiving APs against AIMetS.

#### 2.5.5. Ferritin

Ferritin is a protein and the main intracellular iron storage mechanism. It is synthesized in hepatocytes [175]. Serum ferritin level is a biomarker for assessing the overload of the human body with iron. In addition, elevated ferritin levels are a biomarker of oxidative stress and MetS [176]. An increase in serum ferritin is characteristic of some hereditary metabolic diseases, including familial combined hyperlipidemia and familial hypertriglyceridemia [177].

In an animal model study where rats were given APs (clozapine and haloperidol) for 12 weeks, female rats were found to store iron in the form of hepatic hemosiderin or ferritin granules, which is reflected in higher serum ferritin levels and hemosiderin in the liver, while male rats treated with clozapine increased the level of hepatic hemosiderin [178]. However, another study demonstrated that the concentrations of ferritin in patients with various psychiatric disorders taking APs were inversely proportional to the weight gain induced by risperidone, even after prolonged treatment and despite adequate iron intake. It has also been concluded that low iron stores are associated with poorer response to treatment. Future studies should investigate iron absorption during APs treatment and elucidate the relationship between ferritin levels and AIMetS [179].

#### 2.5.6. Fibrinogen

Fibrinogen is a blood plasma protein. Upon activation of the blood coagulation system, it undergoes enzymatic cleavage by the enzyme thrombin. The resulting fibrin, under the action of active coagulation factor XIII, precipitates in the form of white threads of fibrin-polymer. Together with Plasminogen activator inhibitortor-1 (PAI-1), high plasma fibrinogen levels contribute to the elevated cardiovascular risk, characteristic of people with MetS [180]. Hyperfibrinogenemia has long been regarded as MetS [181]. In contrast to the control group, a correlation between higher levels of fibrinogen and MetS was found in the offspring of hypertensive patients [182].

Patients treated with typical APs had slightly elevated PAI-1 but similar fibrinogen levels compared to patients treated with atypical APs. In addition, the mean plasma levels of fibrinogen and PAI-1 in Sch patients who had taken APs for more than 10 years were slightly higher than those who had taken APs for 10 years or less. Patients taking typical APs and those having used atypical APs for more than 10 years may benefit from periodic assessment of prothrombotic biomarkers for early assessment of the risk AIMetS [183].

#### 2.5.7. Fibroblast Growth Factor-21

Fibroblast growth factor (FGF)-21 is a polypeptide produced predominantly in liver tissues [184]. It has beneficial effects on glucose and lipid metabolism and has high insulin sensitivity [185]. Serum levels of FGF-21 were significantly higher in overweight/obese individuals than in normal or underweight individuals. The level of FGF-21 was positively correlated with obesity, fasting insulin and TG and negatively with LDL-C. An association between FGF-21 and MetS has been found in adults [186] but not in children [187]. Elevated levels of FGF-21 are associated with carotid atherosclerosis, regardless of established risk factors, including adverse lipid profiles and CRP [188].

It is currently proposed that the FGF system is involved in a variety of processes that are likely associated with Sch, and that manipulation of FGF and its receptors results in Sch-associated phenotypes in rodents. It has also been hypothesized that genetic variations in growth factors (FGF and other factors such as BDNF) increase the risk of developing psychiatric disorders. Because these growth factors play a role in the development of the nervous system, they can lead to the subtle changes in brain structure seen in Sch. We know that disruption of FGF signaling can affect dopamine signaling, neuronal proliferation and differentiation in the cerebral cortex. Dopamine disturbances and (prefrontal) cortical dysfunction are associated with Sch and may exacerbate each other. The FGF gene encoding a protein of the same name has been proposed as a candidate gene for Sch and other major mental disorders [189].

#### 2.5.8. Monocyte Chemoattractant Protein-1

Monocyte chemoattractant protein-1 (MCP-1) is considered a key cytokine in the recruitment of monocytes from the blood to early atherosclerotic lesions and plays an important role in atherosclerosis, secreted by adipocytes [190]. MCP-1 levels are higher in patients with MetS and are associated with mild systemic inflammatory response [191]. Elevated MCP-1 levels have also been observed in patients with DM 2 with MetS [192].

In a study in China, MCP-1 and interleukin-8 (IL-8) concentrations were significantly higher in patients with Sch than in controls. In addition, the serum concentrations of MCP-1 and IL-8 in patients were positively correlated with the severity of AIMetS symptoms [193].

#### 2.5.9. Plasminogen Activator Inhibitor-1

Plasminogen activator inhibitor-1 (PAI-1) is a protein of the blood coagulation system. It is a member of the proteins of the serine protease inhibitor superfamily (serpins) [194]. PAI-1 is the main and fast-acting inhibitor of the fibrinolytic system [195] which is sometimes called the prothrombotic adipokine [196]. Elevated plasma PAI-1 is a common feature in MetS patients [197] and is directly related to disease severity [198]. Moderate-intensity exercise [199] and weight loss with a low-calorie diet reduces PAI-1 levels [200].

#### 2.5.10. Retinol-Binding Protein 4

Retinol-binding protein 4 (RBP-4) is a protein that specifically transports retinol in the blood. It is predominantly produced in the liver but is also produced in increased amounts by adipocytes in obesity, which contributes to impaired insulin action [201]. In a cross-sectional study, it was found that levels of RBP-4 gradually increased with an increase in the number of MetS components [202]. Moreover, elevated plasma levels of RBP-4 have been associated with an unfavorable profile of markers of oxidative stress and inflammation [203]. RBP-4 correlates with waist-to-hip ratio or areas of visceral fat [204], and can be used as a predictive biomarker for MetS [205]. A marked decrease in RBP-4 levels after bariatric surgery correlates with a decrease in visceral fat mass and the degree of change in MetS severity [206].

#### 2.5.11. Tumor Necrosis Factor-Alpha

Tumor necrosis factor-alpha (TNF-α) is a pro-inflammatory cytokine that modifies adipose tissue function, influences adipogenesis and is involved in complications associated with obesity [207]. TNF-α levels are elevated in patients with MetS [208].

Increased levels of TNF-α (*p*-value < 0.005) were also found in patients with Sch with an average and high risk of AIMetS compared with a control group [209].

### 2.6. Lipids

Traditional serum or plasma biomarkers from this group include TC, TG and HDL-C (or TC/HDL-C ratio). Dyslipidemia is characterized by a spectrum of qualitative and quantitative lipid disorders which lead to disturbances in the structure, metabolism and biological activity of atherogenic and anti-atherogenic lipoproteins. This process includes an increase in chylomicrons, very LDL (VLDL), LDL, lipoproteins containing apo-B and TG, and low levels of HDL-C [88].

The above indicators also increase with AIMetS. For example, in a study of 82 patients who received atypical APs, 30 patients (36.6%) were diagnosed with secondary DM 2 after 5 years of observation, accompanied by weight gain and increased TG, VLDL and LDL [210].

#### 2.6.1. Oxidized Low Density Lipoprotein

Oxidized LDL has antigenic properties that lead to the formation of antibodies and an inflammatory response which contributes to the formation of atherosclerotic lesions [211]. Oxidized lipoproteins are thought to be associated with cardiovascular risk factors in MetS [212]. Elevated levels of circulating oxidized LDL have been found for different MetS cohorts in various countries [213].

In a study of oxidized LDL levels in patients with Sch, a positive significant correlation was noted between oxidized LDL and IL-27 (r = 0.61, *p*-value = 0.001). Moreover, oxidized LDL can stimulate dendritic cells to produce IL-27, and IL-27 accelerates CHD and MetS through Th1 induction, differentiation and production of pro-inflammatory cytokines. The same paper reported elevated blood levels of IL-27 in patients with Sch, and that APs therapy increases the level of IL-27 in the blood serum, which, in turn, increases the incidence of MetS [214].

#### 2.6.2. Apolipoprotein A1

Apolipoprotein A1 (apo-A1) is the main structural protein found in HDL. This lipoprotein plays a critical role in cholesterol transport by moving excess cholesterol from tissues to the liver. Apo-A1 is usually present in high concentrations in plasma and extravascular tissues. It is synthesized by the intestines and liver. Measurements of apo-AI levels have proven to be very useful in diagnosing and monitoring genetic diseases associated with low HDL-C. Apo-A1 has been found to be able to differentiate patients with CVD [88]. The ratio of apo-B to apo-A1 is higher in MetS and is a predictive biomarker of MetS and preMetS [215]. This ratio has been associated with carotid involvement, intima-media thickness [216] and atherogenesis [217], and is a risk factor for MI in the elderly [88].

The levels of ApoA1 in serum were significantly reduced in patients with Sch receiving APs compared with healthy people (*p*-value = 0.002), while a decrease in serum ApoA1 was also determined in patients with MetS, but these results did not have a high level of significance [218].

#### 2.6.3. Apolipoprotein B

Apolipoprotein B (apo-B) plays an important role in cholesterol metabolism. One apo-B molecule was found in each particle of chylomicron, VLDL and LDL. Apo-B makes up the majority of the total LDL proteins and has two forms: the first is mainly synthesized by the liver and promotes the transport of cholesterol from LDL to various tissues, acting as a ligand for LDL receptors; the second is produced only by the intestines [219] and is involved in the catabolism of chylomicron residues in the liver. Many studies have confirmed that there is a correlation between apo-B containing lipoproteins and the development of atherosclerosis [220]. Apo-B concentrations may be more indicative of CVD than the use of traditional biomarkers. An association between MetS constituents and higher levels of apo-B has also been found [221].

#### 2.6.4. Free Fatty Acids

Free fatty acids (FFA) are an important source of energy for the body. When TGs in adipose tissue undergo lipolysis, FFAs are released into the bloodstream. This process is inhibited by insulin and stimulated by catecholamines, glucagon and adrenal corticoids [222]. FFAs in the blood bind to albumin and are transported to various body tissues. Muscles and the heart use FFA as their main source of energy. The liver oxidizes FFA or forms TG. In addition, FFA, together with cholesterol, are fundamental structural components of the nuclei of LDL-C and VLDL-C, as well as membrane phospholipids [223]. Many studies have associated elevated plasma FFA concentrations with DM 2, obesity and other insulin-resistant conditions [224].

FFAs are involved in the inflammatory process, and elevated concentrations of FFAs can lead to the development of coronary artery disease. Serum fatty acid patterns reflecting low linoleic acid levels and high saturated fatty acids were strongly associated with MetS in individuals with CHD [225]. The higher the concentration of saturated fats, monounsaturated fats and trans fats, the higher the risk of MetS [226]. Fatty acid composition in serum samples can be used not only as a biomarker of fat quality, but also as an indicator of disease risk [227], since fatty acid composition has been associated with metabolic and cardiovascular disease in observational and intervention studies [228]. The erythrocyte fatty acid profile not only shows association with MetS, but also correlates with most of its individual components. It is worth noting that a separate analysis of various trans fatty acids is a more accurate approach to determine their role in the pathogenesis of MetS [229].

There was a significant increase in serum FFA levels in patients with Sch who received long-term APs therapy, especially in groups with obesity, impaired glucose tolerance and DM 2. Elevated serum FFA was positively correlated with fasting blood glucose and insulin resistance [230]. Many other AIMetS studies have shown similar results [231].

### 2.7. Enzymes

#### 2.7.1. Superoxide Dismutase

Superoxide dismutase (SOD) describes a group of metalloenzymes. SOD form the front line of defense against injury mediated by reactive oxygen species (ROS). These proteins catalyze the dismutation of the free radical superoxide anion (O_2_^−^) into molecular oxygen and hydrogen peroxide (H_2_O_2_) and reduce the level of O_2_^−^, which damages cells in excessive concentrations. Thus, SOD is a very important antioxidant defense of the body against oxidative stress [232].

SOD is negatively correlated with MetS components. There are various data on this, with most of them revealing that reduced SOD values are determined in people with MetS [233].

Patients with Sch who did not take APs for 2 weeks had higher levels of SOD, IL-2 and IL-6. At baseline, these SOD increases were associated with higher Positive and Negative Syndrome Scale (PANSS) total scores, and IL-2 increases were associated with lower PANSS positive symptom scores. After treatment, the positive symptoms of PANSS, as well as SOD and IL-2, showed a significant decrease, but IL-6 did not change. The decrease in SOD and IL-2 correlated with the decrease in the total PANSS score [234]. Additionally, a decrease in SOD was determined in the treatment of Sch with risperidone [235]. Thus, one would expect a decrease in the levels of SOD with AIMetS.

#### 2.7.2. Gamma-Glutamyltransferase

Gamma-glutamyltransferase (GGT) is a cell surface protein that promotes extracellular catabolism of glutathione. This enzyme is expressed in many tissues, but mainly in the liver. In serum, GGT is transported mainly by lipoproteins or albumin. Serum levels of this enzyme depend on several factors: alcohol consumption, body fat content, plasma lipoprotein and glucose levels and the use of various medications [236]. In one study, elevated serum GGT predicted the onset of MetS and CVD [237].

An increase in GGT is closely associated with hepatic steatosis [238], and steatosis is associated with MetS [239]. In this regard, high levels of serum transaminases are associated with MetS [240]. An association between elevated serum GGT levels and arterial hypertension [241], as well as an increased risk of DM 2 [242], were also confirmed. Elevated serum GGT largely reflects the presence of ectopic liver fat or secondary inflammation. GGT is used as a sensitive biomarker of increased oxidative stress [243].

In a prospective study, Sch patients (27.2%) treated with APs experienced asymptomatic elevations in GGT levels along with serum transaminases in the first month of the study. After 6 months of treatment, abnormal liver function tests were observed in 22.7% of patients [244].

#### 2.7.3. Glutathione Peroxidase

Glutathione peroxidase (Gpx), i.e., isofremnet GPx3, circulates in human plasma. Defective GPx3 expression in adipocytes is associated with decreased systemic GPx activity in obesity [245]. Serum Se has been found to be associated with MetS components, in particular, HDL-C. Serum Se concentration significantly increased with TC, TG, LDL-C and serum glucose in Taiwanese elderly patients, as well as in Serbian patients with MetS suffering from Sch [246]. A recent study of European patients showed that Se was positively associated with MetS in women but not in men [247]. No associations were found in a Croatian population, but it was found that significantly higher GPx activity was observed in subjects with MetS [248]. In the Japanese study, the MetS group showed a negative correlation of serum Se levels with the monocyte chemoattractant protein-1 (MCP-1). This protein (MCP-1) is critical for the initiation and development of atherosclerotic lesions in MetS [249].

According to a meta-analysis, it was found that GPx was significantly reduced in all patients with Sch, regardless of APs, which, in turn, confirms that GPx will be reduced with AIMetS [250].

#### 2.7.4. Lipoprotein Associated Phospholipase A2

Lipoprotein-associated phospholipase A2 (Lp-PLA 2) has been characterized as a novel inflammatory biomarker that correlates with several components of MetS and atherosclerosis and is a causative agent of CVD [251]. Lp-PLA 2 is predominantly secreted by monocytes and macrophages and oxidatively hydrolyzes modified LDL by cleaving oxidized phosphatidylcholines, generating lysophosphatidylcholines and oxidized FFAs. Numerous prospective and cohort studies have shown that this enzyme is proatherogenic. Lp-PLA 2 is associated with MetS. Increased Lp-PLA 2 activity may be indicative of higher risk. The activity of the enzyme is highly dependent on ferritin, LDL-C and apo-B100 levels. Increased susceptibility to oxidation is manifested by increased levels of ferritin, low levels of HDL-C and reduced levels of antioxidant vitamins [252]. Lp-PLA 2 has a complex role in lipid peroxidation. It was significantly elevated in patients with thickened intima in the carotid arteries compared to those without atherosclerotic plaques [253].

### 2.8. Vitamins

#### 2.8.1. 25-Hydroxyvitamin D

Reduced levels of 25-hydroxyvitamin D (25(OH)D) and increased levels of parathyroid hormone (PTH) have been associated with both MetS and each of its individual components. It is suggested that the decrease in 25(OH)D levels in MetS is due to 25(OH)D binding in body fat [117], and PTH elevation is considered to be a compensatory mechanism for low 25(OH)D levels. Some authors have argued that serum 25(OH)D, but not PTH, was significantly associated with MetS and its components [121].

Also, in women with Sch receiving APs, bone mass decreases; this is associated with drug-induced hyperprolactinemia, which subsequently causes a decrease in the level of 25(OH)D [254].

Data from a population study were confirmed in patients with psychotic disorders and MetS symptoms: the decrease in vitamin D concentration (62.7%) is comparable to the prevalence found in a large survey of people with Sch (65.3%) [255]. Vitamin D deficiency has been associated with an increased risk of MetS, which is consistent with data obtained in the general population [256].

#### 2.8.2. Alpha Tocopherol

Alpha-tocopherol, the main form of vitamin E, acts as an antioxidant vitamin in the human body. Several studies have reported that serum vitamin E concentrations in patients with MetS are lower than in controls, demonstrating an unbalanced serum redox status with reduced lipid antioxidant capacity in MetS [257]. In Chinese women with MetS who received vitamin E supplements, measures of oxidative stress decreased and lipid status improved [258].

## 3. Urinary Biomarkers of Antipsychotic-Induced Metabolic Syndrome

We have identified seven groups of 18 urinary biomarkers: carbohydrates, amino acids, acids, hormones, other organic compounds, metals and other biomarkers.

### 3.1. Carbohydrates

#### 3.1.1. Glucose

Glucose is the most studied urinary biomarker for MetS and AIMetS. The urine in a healthy individual is nearly free of glucose. This may change in patients with DM, where hyperglycemia may enhance filtered glucose and overwhelm the tubular transport capacity for glucose [259]. The normal glucose level in urine is 0–0.8 mmol/L. Glucose in the urine can be determined by the colorimetric method, as well as nuclear magnetic resonance (NMR) spectroscopy. Impaired glucose metabolism is a mandatory risk factor for the development of MetS, as defined by the WHO. Impaired glucose metabolism is associated with insulin resistance, pre-diabetic and diabetic conditions. Elevated urinary glucose has been shown to be a biomarker of insulin resistance [260]. Urinary glucose testing may be a simple alternative or adjunct to blood glucose testing for the diagnosis of MetS in patients with Sch [102]. An increase in body weight and a violation of lipid metabolism may occur within a few weeks after the start of AP treatment. Frequent monitoring of body weight during the first few months of treatment with repeated assessments of glucose and lipid levels may reveal emerging metabolic problems. The sensitivity of determining the level of glucose in urine as a screening test is low (from 21% to 64%), but its specificity is high (>98%). Determinations of glucose levels in urine should be carried out only in conditions of limited resources, when other procedures are not available [261].

#### 3.1.2. Maltitol

Maltitol is a hygroscopic, non-reducing sugar and disaccharide polyol that is used as an alternative to sugar. It is poorly absorbed in the small intestine and has lower insulinemic and glycemic indices and a lower caloric value and sweetening power than sucrose [262]. Maltitol is used as a low-calorie sweetener in chewing gum, ice cream and baked goods [263]. However, an increase in the level of maltitol, as well as glucose, in the urine is a biomarker of insulin resistance [260].

### 3.2. Amino Acids

#### 3.2.1. Aromatic Amino Acids

Aromatic amino acids (AAAs) are formed as a result of proteolysis in the gastrointestinal tract. AAAs include phenylalanine (Phe), tryptophan (Trp) and tyrosine (Tyr). Phe is the metabolic precursor of Tyr via Phe hydroxylase in the liver, which is further metabolized into neurotransmitters such as dopamine, norepinephrine and epinephrine or melanin. Trp is either metabolized via the kynurenine pathway for degradation or used to synthesize serotonin in the gut and brain [264]. In a study involving patients with MetS, AAA were associated with a risk of DM 2 [265]. In a recent study, AAAs were proposed as biomarkers of MetS in urine analyses [260].

#### 3.2.2. Histidine

Histidine (His) is an essential amino acid. It is found in red meat and fish and is present in the body in various forms, including free L-His, N (alpha)-acetylhistidine, His-containing protein and His-containing dipeptides such as like carnosine and anserine. His can be detected in urine using the high performance liquid chromatography method [266]. Reduced levels of His in the urine may be a MetS biomarker and is associated with a violation of the concentration of the endogenous ligands of the imidazoline and α2-adrenergic receptors, which is ultimately associated with episodes of arterial hypertension [260].

#### 3.2.3. Tryptophan

Tryptophan (Trp) is an essential amino acid that is obtained exclusively from food. Trp and its metabolites play an important role in cell growth and maintenance and in coordinating body responses to environmental and dietary signals, in which Trp metabolites serve as neurotransmitters and signaling molecules [267]. Urinary Trp levels were found to be elevated in patients with MetS. A comparison of Trp concentrations in the urine of a control group and patients with MetS showed that it was significantly increased in patients under 60 years of age. Since aging is a risk factor for the development of MetS, the data obtained may recommend Thr as a urinary biomarker of MetS in patients with Sch under 60 years of age [268].

### 3.3. Acids

#### 3.3.1. P-Cresol Sulfate

P-cresol sulfate is produced by the intestinal flora from amino acids. With a decrease in the filtration function of the kidneys, its synthesis increases [269]. Serum P-cresol sulfate concentration increases with deterioration of renal function and correlates with levels of glomerular filtration [270]. Elevated p-cresol sulfate levels in urine signal insulin resistance in patients with MetS [260].

#### 3.3.2. Salicyluric Acid

Salicyluric acidis a glycine conjugate of salicylic acid which forms from acetylsalicylic acid after oral administration. It is hydrolyzed by liver and blood esterases [271], and is the main form in which salicylates are excreted from the body through the kidneys. Elevated salicyluric acid level in urine signal insulin resistance and MetS [260].

#### 3.3.3. 4-hydroxyphenylpyruvic Acid

A type of α-keto acid, 4-Hydroxyphenylpyruvic acid (4-HPPA) is an intermediate in tyrosine metabolism and has a wide range of applications in the food, pharmaceutical and chemical industries. The presence of high concentrations of 4-HPPA in blood and urine could be regarded as an indicator of tyrosinemia [272]. Elevated 4-HPPA levels in urine signal impaired glucose metabolism [260].

#### 3.3.4. Trigonelline

Trigonelline is synthesized from nicotinic acid, which is a catabolite of pyridine nucleotides. It is found in some seeds, but the most trigonelline-rich dietary product is coffee [273]. Approximately 50% of ingested trigonelline is excreted in the urine 0–8 h after ingestion [274]. Trigonelline has been shown to have hypoglycemic, neuroprotective, anti-invasive, estrogenic, anti-cancer and antibacterial effects, as well as anti-caries effects [273]. Decreased urinary trigonelline levels may serve as a biomarker of obesity, dyslipidemia and insulin resistance in Sch patients with MetS [260].

### 3.4. Hormones

#### 3.4.1. Epinephrine

Epinephrine is a catecholamine produced by the adrenal medulla. It is formed from norepinephrine as a result of enzymatic synthesis and accumulates in chromaffin cells. Epinephrine is released into the blood in response to physical or emotional stress. It is involved in the transmission of nerve impulses to the brain, promotes the release of glucose and fatty acids and dilates bronchioles and pupils [275]. With normal renal function, the study of urinary excretion of catecholamines is a reliable method for assessing the state of the sympathetic-adrenal system; 24-h urinary adrenaline excretion specifically reflects adrenal activation [276].

Epinephrine stimulates the hepatic gluconeogenesis and hydrolysis of triglycerides in adipocytes. At the same time, it releases free fatty acids. Urinary epinephrine levels can be determined using high-performance liquid chromatography. It has been shown that complex interactions between the insulin and sympathoadrenal systems can lead to the development of obesity and MetS. Patients with an increase in the number of components of MetS (DM 2, arterial hypertension, dyslipidemia, obesity and/or albuminuria) show lower urinary output of adrenaline [276].

#### 3.4.2. Norepinephrine

Norepinephrine is a neurotransmitter and hormone. It is formed in the sympathetic nerve endings, the adrenal medulla and the CNS, from dopamine [275]. It is involved in the adrenergic regulation of the functions of organs and tissues by the sympathetic nervous system. It acts as a neurotransmitter in the CNS. Twenty-four-hour urinary norepinephrine excretion is a fairly reliable indicator of overall sympathetic activity. The level of norepinephrine in the urine can be determined using the methods of competitive enzyme immunoassay or high-performance liquid chromatography. In Sch patients with an increase in the number of components of MetS (DM 2, arterial hypertension, dyslipidemia, obesity and/or albuminuria) 24-h urinary excretion of norepinephrine may be elevated [276].

### 3.5. Other Organic Compounds

#### 3.5.1. Albumin

Albumin level in urine is an important biomarker of DM 2 and cardiovascular disease. Normally, a small amount of albumin is found in the urine, which is not determined by conventional methods. If the renal glomeruli are damaged, the content of albumin in the urine increases significantly. As a rule, small albumins (microalbumins) are excreted. However, as the disease progresses, larger fractions of albumin are also found in the urine. In this case, analysis shows the presence of protein in the urine. Further development of the disease threatens the general circulation in the kidneys, a decrease in their function and the development of chronic renal failure. The concentration of albumin in urine is measured by semi-quantitative test strips or immunochemical methods: immunonephelometric, immunoturbidimetry and radioimmunoanalysis [277]. Elevated urinary albumin levels have been shown to be associated with the development of DM2 [278]. The WHO considers microalbuminuria as a risk factor for the development of MetS. It has been shown that microalbuminuria is associated with the development of MetS and correlates with the risk of developing arterial hypertension [260]. Microalbuminuria were 2.6 times more abundant in adults with arterial hypertension and DM 2 than in adults without hypertension or DM 2. The prevalence of microalbuminuria was significantly more common in individuals with MetS (36.0%) than in individuals without (5.4%) [279].

#### 3.5.2. Imidazole

Imidazole is a heterocyclic aromatic organic compound. Almost 80% of patients suffering from MetS have high blood pressure. A decrease in the level of imidazole may be associated with disturbances in the concentrations of endogenous ligands of the imidazoline and α2-adrenogenic receptors, which are ultimately associated with episodes of hypertension [260].

#### 3.5.3. Trimethylamine N-oxide

Trimethylamine N-oxide (TMAO) is a small organic metabolite derived from the intestinal microbiota. It has been considered in several studies as a new, potentially important cause of increased risk of developing CVDs [280,281]. The level of TMAO increases after intestinal microbial metabolism of dietary products rich in L-carnitine and phosphatidylcholine, including red meat, eggs and dairy products. It was found that patients with MS had higher levels of TMAO compared to patients without [282]. It was also shown that urinary TOO was associated with risk of CHD [283].

### 3.6. Metals

#### 3.6.1. Cadmium

Cadmium is a common toxic metal. An increase in the level of cadmium in the human body can be caused by a number of factors: cigarette smoking, diet (whole grains, fish and green leafy vegetables), professional activities or living near an industrial area. Studies on the impact of cadmium on adverse health outcomes (e.g., cardiovascular diseases, chronic kidney disease) are controversial [284]. For example, the association between cadmium levels and hypertension depended on whether urinary or blood cadmium levels were assessed [285,286]. The most likely associations were observed when evaluating smokers. In this group, people with the highest urinary cadmium levels had increased chances of developing MetS, as well as low HDL levels. These results were maintained, even after adjustment for dietary factors and serum cotinine levels [284].

#### 3.6.2. Lead

Lead enters the human body orally and is absorbed through the intestines. Cigarette smoke and air pollution are additional sources of exposure. A significant positive association was found between urine lead levels and CVD risk factors, including diastolic blood pressure and mean blood pressure, as well as markers of glucose homeostasis, such as serum glucose, serum insulin levels, evaluation of the insulin resistance homeostasis model, homeostasis model evaluation of β-cell function and BMI [287].

#### 3.6.3. Mercury

Mercury is a highly toxic metal that can accumulate in the tissues of the body. It is used in thermometers, dental amalgam fillings and batteries. Mercury salts may be part of some antiseptic substances and cosmetics (creams, ointments and solutions) [288]. Mercury enters the human body through the lungs when inhaling vapors, through intact skin due to absorption or when eating contaminated food. Consistent data were obtained during the analysis of the relationship between mercury exposure and dyslipidemia. A significant relationship was shown between the amount of mercury consumed and the level of LDL-C in the blood serum. Moreover, a strong negative correlation was found between mercury intake and HDL-C. However, analyses of the concentrations of heavy metals in the urine of individuals suffering from CHD did not reveal a significant relationship between levels of mercury and severity of disease [289].

### 3.7. Other Indicators

#### Potential of Hydrogen (pH)

The urine of a healthy person normally has a pH ranging from 4.5 to 8.0. Usually, urine is slightly acidic (pH from 5.0 to 6.0) [283]. Fluctuations in the pH of urine depend on diet. Changes in urine pH reflect the acid–base state of the blood [275]. Twenty-four urine pH was significantly correlated with serum HDL cholesterol and inversely related to BMI, serum glucose and serum triglycerides in patients with MetS [290].

## 4. Discussion

We reviewed potential blood (Table 1) and urinary (Table 2) biomarkers and their role in the pathogenesis and diagnosis AIMetS in patients with Sch.

**Figure 2:**
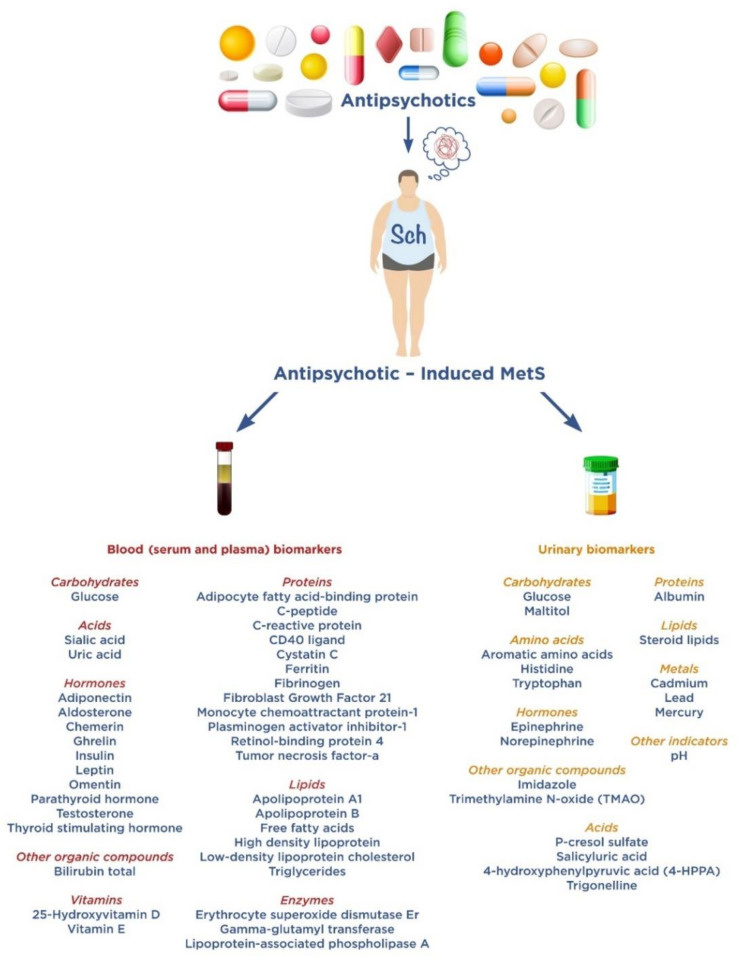
Potential Blood (Serum and Plasma) and Urinary Biomarkers of Antipsychotics—Induced Metabolic Syndrome (AIMetS) in Patients with Schizophrenia (Sch).

Metabolome in patients with Sch determines the individual sensitivity to AP exposure. The metabolome is a complete set of small molecules in the body, comprising peptides, lipids, amino acids, nucleic acids, carbohydrates, biogenic amines, vitamins and minerals, as well as any chemical compounds with which a person is in contact, including food additives, medications, cosmetics and toxins. The formation of the metabolome is influenced by the following: the exposome, i.e., a set of environmental factors influencing the regulation of genes and individual development of organisms; the transcriptome, i.e., a collection of all transcripts synthesized by a single cell or group of cells, including non-coding ribonucleic acid (RNA); the proteome, i.e., a set of proteins of an organism produced by a cell, tissue or organism; and the genome, i.e., a set of hereditary factors [291,292]. Depending on the metabolome, the patient may develop (or not) MetS due the action of drugs, for example APs. The formation of the metabolome is influenced by many factors. We only analyzed the part of metabolome, including uric and serum biomarkers of AIMetS. We can predict and diagnose MetS on the basis of these biomarkers. Figure 2 presents the panel of AIMetS in which we summarize the most significant blood and urinary biomarkers.

We identified three types of AIMetS development based on the absence or presence of AIMetS biomarkers in the blood (plasma or serum) and urine of patients who had received APs therapy for 3 months: definite, probable and possible.

The definite type of AIMetS is characterized by:

- the presence of three or more clinical criteria of MetS according to classification criteria (ATPIII, ATPIII-A or IDF) after 3 or more months of taking APs;
- prolonged use of AP therapy (mono- or polytherapy) for 3 or more months;
- the presence of three or more blood (plasma and serum) and three or more urinary biomarkers AIMetS.

The probable type of AIMetS is characterized by:

- the presence of one to three clinical criteria of MetS according to classifications (ATPIII, ATPIII-A or IDF) after 3 or more months of taking APs;
- prolonged use of AP therapy (mono- or polytherapy) for 3 or more months;
- the presence of one to three blood (plasma and serum) or one to three urinary biomarkers AIMetS.

The possible type of AIMetS is characterized by:

- the absence of clinical criteria of MetS by classification (ATPIII, ATPIII-A or IDF) after 3 or more months of taking APs;
- prolonged use of AP therapy (mono- or polytherapy) for 3 or more months;
- the presence of single blood (plasma and serum) or single urinary biomarkers AIMetS or the absence thereof.

The absence of blood (plasma or serum) and urinary biomarkers in schizophrenic patients within 3 months of starting APs therapy does not exclude the possible development of AIMetS. We assume that re-evaluations of blood (plasma and serum) and urine biomarkers should be carried out (for example, in the ‘probable’ group, once every 3 months, and in the ‘possible’ group, once every 6 months).

**Figure 3:**
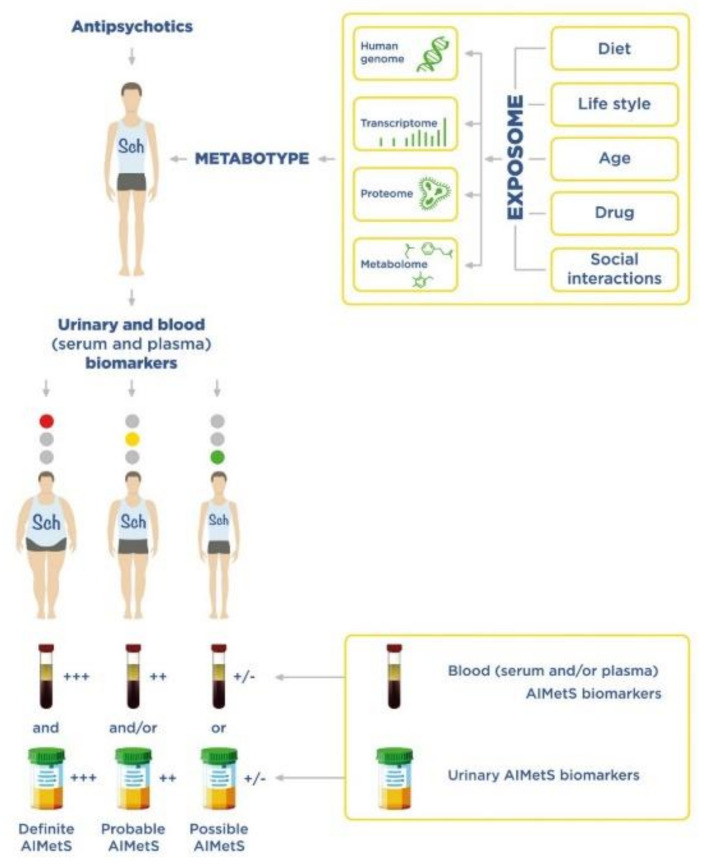
Algorithm of Personalized Approach to the Diagnosis of Antipsychotics—Induced Metabolic Syndrome (AIMetS) in Patients with Schizophrenia (Sch) Based on Blood and Urinary Biomarkers: Definite, Probable and Possible AIMetS.

The prevention of undesirable reactions is especially relevant for patients with SSDs. Taking APs is a long-term, often life-long treatment for this group of patients. The development of MetS leads to the self-cancellation of APs and worsens the quality of life of the patient. There are currently insufficient laboratory and other diagnostic studies in clinical practice to prevent the development of AIMetS. Developments in laboratory research, including molecular-genetic testing, biochemical testing and urinary and blood (plasma and serum) analyses, should be a priority (Figure 3).

## 5. Limitation

This review was devoted to the generalization of potential biomarkers of AIMetS in blood and urine. We emphasized the importance of developing and applying panels of metabolic biomarkers of urine and blood to help ensure timely diagnoses and risk assessments of this ADR of chronic antipsychotic therapy for early prevention in clinical psychiatric practice. This paper consists of two main parts: one dedicated to blood biomarkers and the other to urine biomarkers. Although we have tried to provide a complete list of biomarkers from various studies, we were limited in our ability to describe their roles without further analyses and validation. Undoubtedly, a future meta-analysis is needed to understand the difference between metabolic biomarkers of AIMetS compared to those of MetS in patients with Sch. The authors acknowledge that in preparing this meta-analysis, many potential metabolic biomarkers of AIMetS (especially urine biomarkers) were excluded, since studies were isolated or few in number in some cases. However, in the future, we can expect an increase in research interest in the problem of urine biomarkers of AIMetS.

Another limitation of our review was the lack of analyses of possible differences in the presented metabolic biomarkers of AIMS in female and male patients with schizophrenia. This was partly due to the fact that we did not find similar studies of biomarkers of AIMS in urine. It should be recognized that most potential urine biomarkers and some new blood biomarkers of AIMetS need further study. Nevertheless, we hope to continue studying this issue.

## 6. Conclusions

This systematic review demonstrates that the development of new laboratory diagnostic technologies expands the possibilities of laboratory screening of MetS and AIMetS in patients with mental disorders who take APs for a long time. Metabiotypes of patients with Sch, based on personalized cumulative assessments of blood (plasma and serum) and urinary biomarkers of AIMetS, will make it possible to provide three diagnoses of AIMetS: definite, probable and possible. This approach will optimize strategies for managing patients with Sch and the timing of laboratory blood and urine tests.

## Figures and Tables

**Table 1:** Potential Serum and Plasma Biomarkers of Antipsychotic-Induced Metabolic Syndrome.

Biomarker	Reference Values	Change in MetS	Symptom of MetS	References
***Carbohydrates***				
Glucose	>100 mg/dL	High	Insulin resistance	[32,36,39,40]
***Acids***				
Sialic acid	2–2.33 mmol/L	High	CHD Acute inflammation	[41,43,48]
Uric acid	M 202.3–416.5 µmol/L, F 142.8–339.2 µmol/L	High	Obesity	[50,53,54,59]
***Hormones***				
Adiponectin	0.6–1.33 g/L	Low	Insulin resistance	[64,71]
Aldosterone	25–315 pg/mL	High	AH	[77]
Chemerin	N/A	High	BMI, CHD	[81,87]
Ghrelin	0–100 ng/L	Low	Obesity, BMI	[89,90,91]
Insulin	2.6–24.9 mcIU/mL	High	Insulin resistance	[97,98,101]
Leptin	M 2–5.6 ng/mL, F 3.7–11.1 ng/mL	High	Insulin resistance, leptin resistance	[103,109]
Omentin	N/A	Low	Obesity, endothelial dysfunction	[112,115]
Parathyroid hormone	15–65 pg/mL	High	CVD	[116,118,119]
Testosterone	M 8.64–29 nmol/L (18–55 y.o.), F 0.29–1.67 (18– 55 y.o.) nmol/L	Low	Obesity	[123,127,128]
Thyroid stimulating hormone	0.27–4.2 µIU/mL	High	CVD	[129,130,133]
***Other organic compounds***				
Bilirubin direct and total	2.5–550 µmol/L	Low	Oxidative stress	[138,139,144]
***Proteins***				
Adipocyte fatty acid-binding protein	<6.2 ng/mL	High	Obesity, cardiometabolic disorders	[149,150,152]
C-peptide	1.1–4.4 ng/mL	High	Insulin-related diseases	[159,160,161]
CD40 ligand	N/A	High	CHD	[164,166]
Cystatin C	0.5–1.2 mg/L	High	AH	[168,171]
Ferritin	M 20–250 µg/L, F 10–120 µg/L	Controversial	Oxidative stress	[177,178]
Fibrinogen	1.8–3.5 g/L	High	AH	[180,183]
Fibroblast Growth Factor 21	M 3.6–1021.4 pg/mL, F 65.3–1209.8 pg/mL	High	Obesity, carotid atherosclerosis	[185,186,187,188]
Monocyte chemoattractant protein-1	N/A	High	CHD	[191,192,193]
Plasminogen activator inhibitor-1	N/A	High	CVD	[194,197,199]
Retinol-binding protein 4	N/A	High	Waist-to-hip ratio, visceral fat areas	[201,202,203,206]
Tumor necrosis factor-a	<8.1 pg/mL	High	CHD	[207,208]
***Lipids***				
Oxidized low density lipoprotein	26–117 IU/L	High	Oxidative stress, inflammation	[213]
Apolipoprotein A1	M > 1.2 g/L, F 1.4 g/L	Low	Insulin resistance, dyslypidemia, obesity	[215,218]
Apolipoprotein B	0.6–1.33 g/L	High	Insulin resistance, dyslypidemia, obesity	[220,221]
Free fatty acids	M 8.3–10.9 ng/mL, F 11.4–13.6 ng/mL	High	Insulin resistance	[224,231]
High density lipoprotein	0.7–1.7 mmol/L	Low	Insulin resistance	[88]
Low-density lipoprotein cholesterol	<2.6 mmol/L	High	Dyslypidemia, obesity	[88]
Triglycerides	<1.7 mmol/L	High	Dyslypidemia, obesity	[88]
***Enzymes***				
Erythrocyte superoxide dismutase Er	1200–2000 U/g	Low	Oxidativestress, inflammation	[232,233,235]
Gamma-glutamyl transferase	M 10–71 U/L, F 6–42 U/L	High	Oxidative stress, inflammation	[237,244]
Lipoprotein-associated phospholipase A	<200 ng/mL	High	CVD	[251]
***Vitamins***				
25-Hydroxyvitamin D	30–100 ng/mL	Low	CVD	[121,255,256]
Vitamin E	5.00–18.00 µg/mL	Low	Oxidative stress	[258]

Note: M—male, F—female, AH—arterial hypertension, BMI—body mass index; CVD—cardiovascular disease, CHD—coronary heart disease.

**Table 2:** Potential Urinary Biomarkers of Antipsychotic-Induced Metabolic Syndrome.

Biomarker	Reference Values	Change in MetS	Symptom of MetS	References
***Carbohydrates***				
Glucose	0–0.8 mmol/L	High	Insulin resistance	[260]
Maltitol	None	High	Insulin resistance	[258]
***Amino acids***				
Aromatic amino acids	None	High	DM 2	[265,291]
Histidine	52–162 µmol/mmol	Low	AH	[260]
Tryptophan	0.4–1.4 mg	High	CVD	[268]
***Acids***				
P-cresol sulfate	None	High	Insulin resistance	[260]
Salicyluric acid	None	High	Obesity	[260]
4-hydroxyphenylpyruvic acid (4-HPPA)	None	High	Insulin resistance	[260]
Trigonelline	None	Low	Dyslypidemia, obesity	[260]
***Hormones***				
Epinephrine	0–20 mcg/24-h	Low	Obesity	[276]
Norepinephrine	15–80 mcg/24-h	High	Obesity	[276]
***Other organic compounds***				
Albumin	<30 mg/g	High	AH, DM 2	[260,278]
Imidazole	None	Low	AH	[260]
Trimethylamine N-oxide (TMAO)	None	Low	Obesity	[283,291]
***Metals***				
Cadmium	0.59–0.77 microgram/L	High	AH and low HDL	[284]
Lead	None	High	BMI, insulin resistance	[287]
Mercury	<10 mcg/L	High	Dyslipidemia	[289]
***Other indicators***				
pH	4.6–8.0	Low	Insulin resistance	[290]

Note: None—typically should not be detected in the urine; MetS—metabolic syndrome; AH—arterial hypertension, BMI—body mass index; CVD—cardiovascular disease.
